# Knowledge Management for Fostering Biostatistical Collaboration within a Research Network: The RTRN Case Study

**DOI:** 10.3390/ijerph15112533

**Published:** 2018-11-12

**Authors:** Jae Eun Lee, Jung Hye Sung, Daniel Sarpong, Jimmy T. Efird, Paul B. Tchounwou, Elizabeth Ofili, Keith Norris

**Affiliations:** 1 Research Centers in Minority Institutions Translational Research Network Data Coordinating Center, Mississippi e-Center, Jackson State University, 1230 Raymond Rd., Jackson, MS 39204, USA; paul.b.tchounwou@jsums.edu; 2 Department of Biostatistics and Epidemiology, College of Public Services, Jackson State University, 350 W. Woodrow Wilson Drive Jackson Medical Mall, Suite 301, Jackson, MS 39213, USA; jung.h.lee@jsums.edu; 3 Center for Minority Health and Health Disparities Research and Education, Xavier University, 1 Drexel Drive, New Orleans, LA 70125, USA; dsarpong@xula.edu; 4 Center for Clinical Epidemiology and Biostatistics (CCEB), School of Medicine and Public Health, the University of Newcastle (UoN), Callaghan, NSW 2308, Australia; jimmy.efird@stanfordalumni.org; 5 Clinical Research Center & Clinical and Translational Research, Morehouse School of Medicine, 720 Westview Drive, Atlanta, GA 30310, USA; eofili@msm.edu; 6 Department of Medicine, David Geffen School of Medicine, UCLA, 911 Broxton Ave, Room 103, Los Angeles, CA 90024, USA; knorris@ucla.edu

**Keywords:** strategic approach, research collaboration, knowledge management, research network, capacity building, RTRN

## Abstract

*Purpose*: While the intellectual and scientific rationale for research collaboration has been articulated, a paucity of information is available on a strategic approach to facilitate the collaboration within a research network designed to reduce health disparities. This study aimed to (1) develop a conceptual model to facilitate collaboration among biostatisticians in a research network; (2) describe collaborative engagement performed by the Network’s Data Coordinating Center (DCC); and (3) discuss potential challenges and opportunities in engaging the collaboration. *Methods*: Key components of the strategic approach will be developed through a systematic literature review. The Network’s initiatives for the biostatistical collaboration will be described in the areas of infrastructure, expertise and knowledge management and experiential lessons will be discussed. *Results*: Components of the strategic approach model included three Ps (people, processes and programs) which were integrated into expert management, infrastructure management and knowledge management, respectively. Ongoing initiatives for collaboration with non-DCC biostatisticians included both web-based and face-to-face interaction approaches: Network’s biostatistical capacities and needs assessment, webinar statistical seminars, mobile statistical workshop and clinics, adjunct appointment program, one-on-one consulting, and on-site workshop. The outreach program, as a face-to-face interaction approach, especially resulted in a useful tool for expertise management and needs assessment as well as knowledge exchange. *Conclusions*: Although fostering a partnered research culture, sustaining senior management commitment and ongoing monitoring are a challenge for this collaborative engagement, the proposed strategies centrally performed by the DCC may be useful in accelerating the pace and enhancing the quality of the scientific outcomes within a multidisciplinary clinical and translational research network.

## 1. Background

Research which incorporates perspectives from ethnically diverse researchers across disciplines into complex scientific problems can facilitate innovative and insightful solutions in a collaborative manner. Activities are performed more effectively by building on and integrating research resources, knowledge, and abilities. However, conflicts caused by socio-cultural differences across disciplines and varied priorities and approaches, coupled with an absence of communicating technologies have hindered many collaborative efforts. Despite the benefits that such novel collaborative tools may bring, communication also has been impeded by organizational obstacles (e.g., the different structures and rewards within and between research entities) and lingering persistence of many funding agencies and review groups for single-discipline, single-center projects. However, Thagard [1] indicated that today’s highly specialized disciplines make it difficult for a single researcher or a group of researchers in a single field to solve a problem that requires cross-disciplinary knowledge. The challenges and opportunities for research collaboration have long co-existed. In the last decade, there has been increasing recognition of the importance of collaborative research and consequently, sponsorship for research networks by funding agencies has increased [2,3].

Modern research is characterized as “greater interdisciplinarity” and “more networking and collaboration” [4]. The World-Wide Web (WWW) and supporting technologies have made it possible for geographically separated researchers to collaborate more seamlessly in achieving a common goal by sharing ideas, information, and work in a synchronous and asynchronous manner. Since the rise of the internet, research collaboration has increased, a variety of research network models have been developed, and scientific productivity has flourished as a result of the collaboration [5,6,7,8]. However, it is still questionable that substantive collaboration can be achieved in a research network because of the traditional lack of a collaborative culture and mutual distrust. The quantity of collaboration in terms of the number of collaborators may not be a significant predictor of scientific outcome. Lee and Bozeman [9,10,11] suggested that “collaborative strategy” is one of the significant factors impacting publishing productivity. Therefore, it is important to develop effective strategies to exploit the potential benefits of collaboration [12].

While the intellectual and scientific rationale for such interdisciplinary collaboration has been articulated [13] and there is an increasing interest and activity in forming research networks [14,15], a paucity of information is available on the specific efforts that need to be expended by foundations to establish and support these innovative interdisciplinary endeavors. Statisticians are key contributors to the research network since they help investigators across all aspects of research (e.g., design, randomization, analysis, interpretation, and publication and presentation of research findings). Therefore, statistical capacity building is an important ongoing component of a research network. As part of the endeavor to provide the evidence to support biostatistical capacity-building in a research network [16], the current study aims to leverage lessons learned from the NIH funded Research Centers in Minority Institutions Translational Research Network (RTRN) data coordinating center (DCC) to: (1) develop a conceptual model for strategic approach to facilitate collaboration driven by the biostatisticians in the Network; (2) describe collaboration engagement performed by the Data Coordinating Center (DCC); and (3) discuss potential challenges and opportunities in engaging the collaboration.

Ultimately, this study is intended to provide an important template in an organized manner based on a novel framework for an early staged research network. This entails fostering collaboration among statisticians at various levels of training and expertise and at different intensive research environment, while optimizing scientific productivity.

### RTRN as the Environment of the Proposed Model

This proposed collaboration model will be applied within a research network designed to reduce health disparities: RCMI Translation Research Network (RTRN). The RTRN is a research network of 18 Research Centers for Minority Institutes (RCMIs) formed to foster collaborative efforts. Its underlying mission is to facilitate innovative multi-, inter-, and trans-disciplinary research and generate evidence-based knowledge and techniques for the diagnosis, prevention, and treatment of diseases outlined in the Healthy People 2010 priorities for improving the nation’s health and reducing health disparities [2]. The RTRN has focused on highly structured multi-site clinical studies/trials which target minority populations at minority institutions, ultimately generating results which translate to better health of the general public. Unlike other research networks such as CaBIG (Cancer Biomedical Informatics Grid) and BIRN (Biomedical Informatics Research Network), RTRN is the first academic-based national research network to specifically address, using hypothesis-driven approaches, the problem of health disparities across the continuum of basic-clinical community investigations. This is accomplished through an integrated network of minority health profession doctoral degree granting schools that have trained over 25 percent of minority physicians and over 50% of minority biomedical and health science PhDs in the USA [17]. The RTRN was established with the principal goal of creating a framework for effective collaborations both within RCMI institutions and with larger research-intensive institutions or organizations. The intent of collaborative relationships was to support partnered research and promote access to information and resources that move the entire research community toward a greater understanding of the tools needed to reduce health disparities to achieve health equity for all Americans. By linking the researchers focused on specific disease conditions (e.g., diabetes, cardiovascular disease) into “clusters” through internet-mediated virtual meeting places or “cyber workspaces”, the cluster system will help researchers from a broad range of disciplines work together more effectively and overcome the barriers imposed by space and time [2]. This is critical to enable the collection, integration, and sharing of large volumes of data from a broad range of data types (e.g., laboratory results, biological samples, psychosocial surveys, electronic health records) to improve the quality of translational research and to fill gaps in the dearth of existing data on minority populations.

Finally, the investment in a high level of computer-based infrastructure supports not only communication tools, but the conduct of multisite clinical trials RTRN supports two widely used clinical research data management systems - Redcap and Oracle Clinical, as well as analyses of large secondary databases and population genetics studies, computational modeling and data mining. These analyses and associated research study designs require a substantive effort from the RTRN biostatistical team. The details on the RTRN are described elsewhere [2,17].

## 2. Components of the Proposed Model: 3 Ps

**Figure 1:**
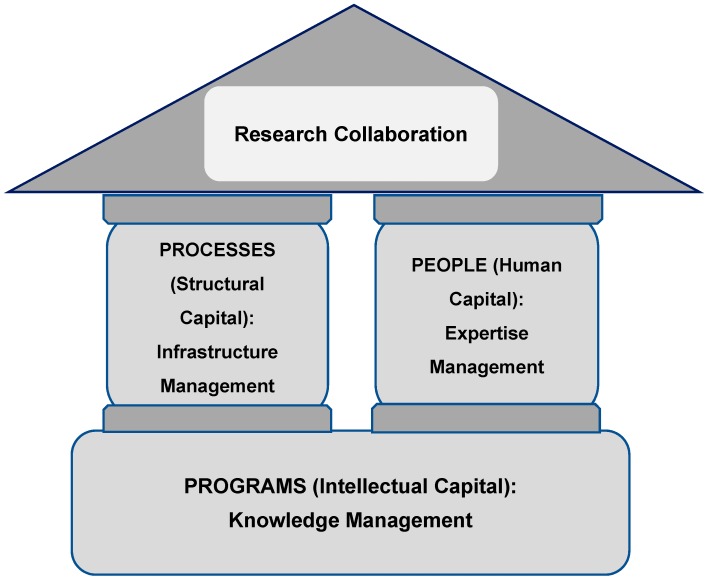
Key Components of the Strategic Approaches.

The components that support the proposed model include the “3 Ps”: people, processes and programs (Figure 1). The *program* is defined as all planned activities to develop and harness Network statisticians’ intellectual capital consisting of both human and structural capital. It may include face-to-face and web-based programs such as statistical webinars, mobile statistical workshops and clinics, adjunct appointment program, one-on-one consulting, on-site workshops, statistical programing courses, and statistical programming certification program. The intellectual capital will be developed through knowledge management. Human capital which resides in the *people* (biostatisticians) of the Network is composed of the skill, talent, experience, knowledge, know-how, and expertise of the Network biostatisticians. It can be described as a Network’s collective statistical capability to best support the Network’s clinical trials/studies. Expertise management skills will be applied for developing human capital. Structural capital related to *processes* can be thought of as the Network’s infrastructure necessary to perform collaborative activities. It includes networking tools (internet, intranet, workspace, Wiki space, listserv), policies and guidelines (Standard Operating Procedures (SOPs), memorandum of understanding (MoU), leadership plan), and data (repository system, data to be shared, and data analysis tools). A strategic approach for developing structural capital will encompass infrastructure management. The approach and potential action plan for the development of the model are summarized in Table 1.

## 3. Strategic Approaches for Collaboration

The DCC has already been involved in various initiatives for collaboration with non-DCC biostatisticians to support the research projects initiated by Network scientists, which may serve as a guide for a success of the proposed collaboration model. A key collaboration strategy that the DCC has applied was to prepare an environment within which participants feel free to share ideas and information and to conduct collaborative work.

### 3.1. Expertise Management

Expertise management is a specialized system or approach that focuses on capturing and continually recording what people (“biostatistical experts”) in the Network know (“expertise”) and making this expertise available to Network researchers/investigators. Utility of this Expertise Management will facilitate match-making between the RTRN statisticians and the researchers/investigators to support investigator-initiated research. It is not simply a document retrieval or file management system but rather an “expert system” that provides diagnostic or problem solving functionality for the conduct of research. The DCC is also currently involved in several Expertise Management Initiatives (See Table 2). The core processes that we have applied to engage expertise management include: (1) identifying the experts, (2) describing the expertise; (3) providing an expertise matching mechanism; and (4) collaborating with the matched expertise for the conduct of research project.

#### 3.1.1. Capacity Assessment

Although a variety of techniques are already developed for identifying experts and expertise [18,19], we started with conducting a survey for assessment of the Network’s Biostatistical needs and capacities [16]. Through this assessment, the RTRN biostatistician working group database was developed, in which 52 clinical and genetic statisticians were inventoried within the RTRN system. The database included contract type (full/part time), academic degree, years in experience as a biostatistician, expertise, and the years of experience using the analytical tools. Among the 52 biostatisticians, 84% are fulltime employees and 53% hold a doctoral degree. They have approximately 13 years of job experience [9.4 years (SD = 8.4) for masters; 15.0 (SD = 7.2) for doctoral statisticians]. RTRN biostatisticians use a total of 22 different types of statistical software. Statistical Analysis System (SAS, SAS Institute Inc., Cary, NC, USA), Statistical Package for the Social Science (SPSS, SPSS Inc., Chicago, IL, USA) and STATA (StataCorp LP., College Station, TX, USA) are the most frequently used statistical software. About 88% of Network statisticians use SAS as their major analytical software which requires advanced programming skills.

#### 3.1.2. Web Search

In order to increase utility of the secondary data that the DCC possesses, we conducted a web-search to identify potential users of the data. We identified 235 RCMI potential investigators within the Network in the research areas of cardiovascular disease, renal-related disease, diabetes, health science research, pulmonary, social psychology, nutrition, physical activity, obesity and social work.

#### 3.1.3. Key Research Team Meeting

Despite the importance of on-going expertise management for a research network, identification of the expertise has largely relied on paper-pencil method or web-search which does not allow feedback and dialog with experts through face-to-face contact. The outreach program (detailed below) that the DCC performed allowed us to identify detailed expertise of key research teams in each member institution through dynamic interaction.

### 3.2. Infrastructure Management (IM)

IM is the managerial action to identify, maintain, upgrade, and place the structural capital such as policies, processes, equipment, data, network tools, and data analysis tools, for attaining the goal of collaborative biostatistical activities within the Network. Infrastructure management generally seeks to: (1) reduce duplication of effort; (2) ensure adherence to good clinical practice standards; (3) enhance the flow of information throughout an information system; (4) promote adaptability necessary for a changeable environment; (5) ensure interoperability among biostatisticians from DCC and non-DCC; and (6) maintain effective policies and practices. DCC engagement for Infrastructure Management included research networking tools, policies and guideline, and data infrastructure (See Table 2).

#### 3.2.1. Research Networking (RN) Tools

These are web-based tools to communicate with Network biostatisticians, share research related data, and post opinion/experience/knowledge in statistics related issues. RN tools are expected to facilitate the development of collaborations to address new or existing research challenges through the rapid discovery and recommendation of researchers, expertise, and resources [20,21]. The DCC created a designated space on the DCC portal and is utilizing a Wiki site for the study support where statistical resources can be shared. Resources include technical notes, SAS codes and other statistical resource links. Statisticians also will have the opportunity to post questions on the space/site and the statisticians within the Network will be notified by email through a listserv allowing each to respond to the posting. While the Wikis have enabled the study sites to quickly share and post non-regulated information, regulated information is being shared through portal workspace. Communication tools including web conferencing, video conferencing, and email listserv also are being utilized for the collaboration, which enable the Network statisticians to participate in bi-directional communication flow. As evidenced in the previous studies [22,23] which proved the significant effect of networking tool on the performance, this approach is expected to reduce the learning curve for various statistical techniques and increase the expertise within the network. In addition, the DCC plans to utilize the infrastructure to support biostatistical collaborations and communication across the network: eagle-i (Resource Discovery Networking system), RTRN website, and Profiles (Research Networking system).

#### 3.2.2. Policies and Guidelines

Statistical collaboration will be carried out in accordance with Good Clinical Practices as interpreted in the ICH Harmonized Tripartite Guideline. All procedures for the statistical collaboration are governed by DCC standard of process (SOPs) on: Statistical Analysis Plan (SAP); Randomization; Blinding; Code-Breaking; Clinical Study Report; and Statistical Quality Control. Participating statisticians will take training on SOPs before starting collaborative work. To ensure compliance, statistical outputs/outcomes jointly produced are subject to audit by our quality control committee. Additionally, MOU, pre-specified written leadership plan/role assignment among Network biostatisticians for the specific research support, and SOP on collaboration among Network biostatisticians will be prepared and applied for the collaborative projects.

#### 3.2.3. GIS Data Capture App

The RTRN DCC Web-based GIS application was developed to achieve the goal of procedural standardization for a multi-site study. This application was developed using publically accessible resources from US Census Bureau, Yahoo, Google, MapQuest, Microsoft, Street Smart WalkScore, and ESRI (ArcGIS, Esri, Redlands, CA, USA). Functions of the GIS application include geocoding, geo-referencing, abstracting US Census data, and deriving GIS variables for fast-food restaurant density, accessibility to health facilities and walkability [24].

#### 3.2.4. Data Infrastructure

Data-related infrastructure that the DCC has utilized for the collaboration with Network biostatisticians includes the data itself, data sharing tools, and data analysis software. The DCC has procured various large datasets to support studies for generating hypotheses and resolving issues during the conduct of trials. The large datasets include the Jackson Heart Study (JHS) which is the largest single-site cohort study to prospectively investigate the determinants of cardiovascular disease among African Americans; the National Health and Nutrition Examination Survey (NHANES) which is a program of studies designed to assess the health and nutritional status of adults and children in the United States where interviews and physical examinations are combined; and the Behavioral Risk Factor Surveillance System (BRFSS) which is the world’s largest, on-going telephone health survey system, tracking health conditions and risk behaviors in the United States yearly since 1984. The DCC has routinely utilized these datasets to address study questions. In addition, data generated through surveys that the DCC supports also are utilized for collaboratively developing manuscripts and reports. All data are formatted into SAS data format and shared through access-regulated virtual workspace. A server-based SAS program allows collaborative biostatisticians to remotely access analytical tools.

### 3.3. Knowledge Management

This involves the planning, organizing, directing, and controlling of the programs to boost the Network’s biostatistical intellectual capital. It includes all programs to capture and disseminate biostatistical knowledge across the Network. The programs address an important means for building research capacity: training and education [25]. Ultimately, participation in such programs may lead to new professional networks and access to social capital [26]. Social network development through the programs may be an important opportunity for biostatistical capacity-building of the Network. Although there exists limited published information on research training programs for biostatistical capacity-building in a research network, the DCC is currently involved in several interesting Knowledge Management Initiatives for biostatistical capacity-building: Mobile Clinic Collaborative Exchange Program, seminars/workshops/consulting, and partnership (see Table 2).

#### 3.3.1. Biostatistical Mobile Clinic Collaborative Exchange Program

The purpose of the outreach is to showcase DCC biostatistical capacity and to foster collaboration for future projects within the Network. The clinic and outreach tours are designed to stimulate exchange of research ideas between the DCC biostatistics core and Network institutions in order to: (1) identify the statistical needs of RTRN, (2) disseminate information about DCC’s statistical expertise and capacity, and (3) identify future research projects that the DCC and the RTRN institution(s) can pursue jointly. A team of three senior statisticians from the DCC visited three geographic areas (comprising 5 RCMIs) from 23 May to 10 June 2011 and interacted with a total of 129 investigators who participated in this program. The Mobile Biostatistical Clinics took “support” out into the scientific community and helped to minimize some of the barriers of communication between statisticians and researchers. A collaborative work with Network and external biostatisticians is planned to implement the continuous outreach program.

#### 3.3.2. Webinar Series/Workshop/Consulting

The DCC has also organized various training programs including webinar series and statistical workshops and one-on-one statistical consulting at RCMI international symposia where most Network statisticians convened. The DTCC provided 4 webinar seminars, 3 on-site workshops and on- and off-site consulting programs. More than 520 RTRN investigators and students have benefited from these programs.

#### 3.3.3. Partnership

During the last years the DCC Biostatistical team has been very instrumental in training and mentoring junior biomedical investigators and collaborating with scientists within and outside RTRN. The Division has built a partnership with member institutions in organizing/implementing training programs. The DCC biostatistical team co-hosted a joint statistical workshop with the Puerto Rico Clinical and Translational Research Consortium at the University of Puerto Rico, 14–16 November 2011. A total of 8 sessions were provided by the DCC statistical team; 16 to 35 scientists and students attended each session.

#### 3.3.4. Adjunct Statistician Program

This program is designed to enable the DCC to harness the niche expertise within the network in a coordinated fashion. Adjunct members are individuals who provide volunteer time and expertise to the DCC. These individuals’ time has been spent participating in research projects, developing publications, developing research grants or other activities (workshops or seminars). There are mutual benefits of the program. The adjunct statistician receives co-authorship of publications in which for he/she contributes and gains experience working with RCMI investigators. The DCC benefits by supplementing insufficient biostatistical personnel resources without the need to hire addition FTEs.

#### 3.3.5. Jackson Heart Study Vanguard Center

As a Jackson Heart Study Vanguard Center, the DCC has helped Network investigators including biostatisticians generate scientific output by providing such customized services for secondary data as preliminary analyses for feasibility, data-mining, data workshops and idea sharing meetings, profiling potential investigators for team building, providing data information and query tools through the website, helping develop manuscript proposals, and providing data analyses for publication. A total of 300 investigators/biostatisticians attended workshops and/or received booklets designed to help potential users better understand the data structure and how to access the data. The DCC provided two online and five onsite workshops, developed/distributed more than 200 copies of the booklet and posted instructions on the RTRN website to facilitate the data utility. A total of four manuscript proposals, 11 presentations, nine manuscript developments (seven published) and two R01 and two small (R03 and R21) grant proposals were supported related to the JHS data [27]. The above mentioned strategic collaboration engagement performed by the DCC is summarized in Table 2.

## 4. Discussion

The proposed collaborative model which will be supported by the various strategic approaches may be successful in enhancing the collaboration among biostatisticians across the Network and develop biostatistical intellectual capital of the Network. The programs that the DCC developed and implemented for Network biostatistical capacity-building are expected to greatly contribute to the model success. The DCC already has been involved in various initiatives for collaboration with non-DCC biostatisticians to support the research activities within the Network. Networking tools that have been used for the multi-site clinical trial supports, experience on collaboration with non-DCC statisticians and investigators, know-how on implementation of the collaborative programs, etc. may be excellent capital to help the proposed collaboration model accomplish the expected outcomes.

One of the important considerations in developing RTRN biostatistician-driven engagements was to facilitate communication between statisticians and scientists since good communication between statisticians and scientists who are involved in this interdisciplinary interface is important [28]. Optimal communication is the mutual recognition of the complexity of the applied research problem in relation to the statistical possibilities and restrictions. Webinars were designed for the scientists to gain scientific and statistical knowledge and confidence in order to be able to choose appropriate statistical methods for a research project. Key research team meetings were to communicate with a common language to identify major methodological issues in the active projects and to make the statistical theories and approaches understandable and relevant to the scientist’s own field of interest. However, it is still questionable if substantive communication among entire participants throughout the network is accomplished.

Collaboration programs that the DCC has provided were to prepare an environment within which participants feel free to share ideas and experience. However, information flow has been unidirectional from the DCC to each RCMI site. The DCC has acted as a major provider of information as well as coordinator of the flow of information. The proposed collaboration model is designed to obtain maximum outcomes when bidirectional information flow is promoted. Further efforts should be placed on involvement of site biostatisticians in the collaborative activities.

Since the RTRN is based on an internet-mediated virtual working environment, the DCC initiatives for the biostatistical collaboration encompassed web-based programs (i.e., webinar seminar series, web-based consulting and research support, etc.). Although the web-based information flow allows minimizing the physical distance that the collaborators should travel for collaborative work, it has a disadvantage that the face-to-face dynamic interaction is not possible. According to Beckmann’s model [29], which assumed face-to-face communication, the likelihood of two researchers collaborating decreases as the physical distance between them increases. Despite the positive impact of emerging communication technologies on scientific research [30,31,32], Lee and Brownstein, et al. [33] found that physical proximity is still an important predictor of research collaboration. The DCC did not disregard the advantages of face-to-face dynamic interaction. The DCC, therefore, developed an integrated model and implemented various on-site programs such as workshops and one-on-one consulting program at the RCMI International Symposium in which most member statisticians and investigators convened. Those programs enabled maximizing the advantages of the face-to-face interaction while optimizing cost for travel.

The mobile workshop program based on face-to-face interaction not only enabled optimizing the total distance that program participants needed to travel since only a minimum of necessary people were deployed to participating sites but also made it possible to take full advantages of the face-to-face interaction. The outreach program entitled the “Biostatistical Mobile Clinic Collaborative Exchange Program” was an “one-for-all” tool for expertise management and needs assessment as well as knowledge exchange. During the mobile clinics, the various expertise and research needs of each institution were identified through face-to-face dynamic discussion. The identified research needs that each research unit wanted to be supported from the DCC included senior level support for grant and manuscript development; hosting database systems; high quality research administration; advertising capabilities of institutional research units to the local community; and match-making between investigators and RTRN research units. Statistics-related requests from the Network, especially for grant proposal and manuscript development, have doubled compared with the prior year since the outreach program was implemented. In addition to achieving the planned purpose, the mobile clinics were successful in identifying investigators’ needs for DCC services as well as identifying existing expertise and resources in the field. Since the outreach program was given in 2010, increased yearly service requests were received from RCMIs where outreach program was given (from 2.3 before 2010 to 16 after 2010) than those where it was not given (from 5.7 to 16). Ultimately, increased request generated by this outreach provided increased opportunities for Network statisticians to be involved in the projects. Therefore, the outreach program and web-based programs that the DCC have developed and implemented for the Network biostatistical capacity-building may be an important knowledge management strategy which will lead to success of the proposed collaboration model.

However, it is important to note that there may be some challenges for successful collaboration within the research network. First, although research networks have led a change in research culture [34], fostering a partnered inter-institutional network culture is still difficult [35], because in the competitive marketplace, each entity develops the sense that they are in competition with the other member in the network. There also is the “What is in it for me” syndrome. To overcome these barriers the model will have to have processes and procedures that articulate a fair, equitable and transparent collaboration. There will need to be relationship building. Approaches that have worked in the past have been the DCC Mobile Biostatistical Clinics and one-on-one consultation sessions hosted at a number of RTRN member institutions. These activities have established trusted relationships and provided opportunities to conduct needs assessment and asset mapping. Continuation of these activities and web-delivery seminar series should foster network culture. The DCC also will have to solicit the assistance of the RTRN Steering committee, RCMI PIs/Program Directors, lead statisticians at the member institutions, and the communication Division of the DCC to develop this sense of trusted community and the commitment to support each other resonate through the network.

Second, senior management commitment may be critical in promoting members involvement and culture change. Previous studies have suggested that strategic leadership from the top can promote involvement of members in planned programs [36,37]. Personal involvement of the Network’s senior management may promote the involvement of biostatisticians in the biostatistical collaboration engagement. Senior management commitment also may be one of the most important and vital principles in a Network’s culture change for collaborative work [38,39]. In this collaboration engagement, senior management should be committed to creating an organizational climate for collaborative work and mutual trust. Thus, this can be achieved with senior management commitment in training Network biostatisticians and giving them opportunities to be responsible for the collaborative work and development and dissemination of the biostatistical knowledge. Senior management also needs to develop measures to enhance the motivation of Network biostatisticians for biostatistical collaborator activities.

Lastly, though the proposed model has great potential and prospect, it is important to monitor and evaluate its success on the regular base. Although it is a challenge to develop metrics in research network, the evaluation plan should include metrics that must be measurable, explicitly defined and customizable [40]. Ongoing monitoring has been previously used to detect a change in outcomes so the process can be examined, reinforcing beneficial practices and eliminating factors that degrade performance [41]. The proposed performance and process indicators and benchmarks will be used to accomplish this end. This phase of the implementation of the conceptual model could be equated to a Phase IV Clinical Trial in drug development (i.e., post-marketing surveillance). The surveillance activities (monitoring and evaluation) afford the DCC and RTRN to implement corrective measures where and when needed and to refine the processes to accomplish the desired outcomes of: (1) optimized efficiency, (2) strengthened collaboration, (3) promoted niche-oriented culture, (4) leveraged scientific output across the network, and (5) strengthened biostatistical capacity across the network.

Overall, several lessons were learned from the team’s experiences that were key to developing the strategy of engagement. For example, interacting with researcher across various disciplines promoted a better understanding and each other’s expertise and approaches to problem solving. In several cases, ideas for future collaborative projects emerged during group conversations among participating researchers. Contacts also were developed for junior level investigators and graduated students to pursue research opportunities at other Network sites. The steps developed from the lessons provide a better understanding of the links for improving upon communication throughout the Network.

## 5. Conclusions

While several challenges remain, the proposed model based on previous work and experience, may be useful in accomplishing the scientific outcomes within the multidisciplinary research network. We believe this proposed model can be an important template for research network biostatistical cores in: providing statistical support for clinical trials or studies in a network; fostering collaboration among statisticians at various levels of training and expertise and at different degree of research environment; and leveraging the niches of the various biostatistical units to promote rewarding collaboration within the Network, while optimizing scientific productivity.

## Figures and Tables

**Table 1:** Strategic Approaches and action plan per component.

Components		Strategic Approaches	Available Plan
People	Human capital	Expertise Management	Inventory, profile management, ongoing survey, match-making tool, etc.
Processes	Structural capital	Infrastructure Management	Networking infrastructure (internet, workspace, Wiki, listserv, etc.); policy and guideline infrastructure (MOU, SOP, leadership plan, etc.); data (repository system, data available, network based analytical tools/
Programs	Intellectual capital	Knowledge Management	adjunct appointment, mobile workshop and clinics, webinar seminars, one-on-one consulting, online and on-site workshop, statistical programing course, certification program, etc.

**Table 2:** Strategic Collaboration Engagement Performed by the DCC.

Approach	Engagements
Expertise Management	Capacity Assessment: conducted survey for assessing the Networks biostatistical needs and capacities. RTRN biostatistical working group database created through the survey.
	Web search: identified 235 RCMI investigators who will be user of the data that DCC possesses.
	Key Research Team Meeting: this outreach program allowed DCC to identify detailed expertise of key research teams in each member institution through dynamic interaction.
Infrastructure Management	Research Networking (RN) Tools: to communicate with Network biostatisticians, share research related data, and post opinion/experience/knowledge in statistics related issues.
	Policies and Guidelines: All procedures for the statistical collaboration were governed by DCC standard of process (SOPs). MOU, pre-specified written leadership plan/role assignment among Network biostatisticians for the specific research support, and SOP on collaboration among Network biostatisticians were developed. GIS Data Capture App: The RTRN DCC Web-based GIS application was developed to achieve the goal of procedural standardization for a multi-site study
	Data Infrastructure: Data-related infrastructure that the DCC has utilized for the collaboration with Network biostatisticians includes the data itself, data sharing tools, and data analysis software.
Knowledge Management	Biostatistical Mobile Clinic Collaborative Exchange program: a total of 129 investigators participated in this program. The Mobile Biostatistical Clinics took “support” out into the scientific community and helped to minimize some the barriers of communication between statisticians and researchers.
	Seminar Series/Workshop/Consulting: The DCC provided four webinar seminars, three on-site workshops and on- and off-site consulting programs. More than 520 RTRN investigators and students have attended these programs.
	Partnership: DCC has built a partnership with member institutions in organizing/implementing training programs.
	Jackson Heart Study Vanguard Center: As a Jackson Heart Study Vanguard Center, the DCC has helped Network investigators including biostatisticians generate scientific output by providing such customized services for secondary data as preliminary analyses for feasibility, data-mining, data workshops and idea sharing meetings, providing data information and query tools through the website, helping develop manuscript proposals, and providing data analyses for publication.
